# Management of physical and psychological trauma resulting from motor vehicle crashes in Australian general practice: a mixed-methods approach

**DOI:** 10.1186/s12875-024-02421-5

**Published:** 2024-05-16

**Authors:** Carla Bernardo, Elizabeth Hoon, David Alejandro Gonzalez-Chica, Oliver Frank, Sean Black-Tiong, Nigel Stocks

**Affiliations:** 1 Adelaide Medical School, The University of Adelaide, 115 Grenfell St, Level 8, Room 817.01, Adelaide, SA 5000 Australia; 2 Adelaide Medical School, School of Public Health, The University of Adelaide, 115 Grenfell St, Level 8, Room 818.01, Adelaide, SA 5000 Australia; 3 Adelaide Medical School, Adelaide Rural Clinical School, The University of Adelaide, 115 Grenfell St, Level 8, Room 811C.02, Adelaide, SA 5000 Australia; 4 Adelaide Medical School, The University of Adelaide, 115 Grenfell St, Level 8, Room 817.09, Adelaide, SA 5000 Australia; 5 Adelaide Medical School, The University of Adelaide, 115 Grenfell St, Level 8, Room 817, Adelaide, SA 5000 Australia; 6 Adelaide Medical School, The University of Adelaide, 115 Grenfell St, Level 8, Room 823.01, Adelaide, SA 5000 Australia

**Keywords:** Traffic accidents, Injuries, Chronic pain, Depressive symptoms, Anxiety

## Abstract

**Background:**

In Australia, motor vehicle crashes (MVC)-related health data are available from insurance claims and hospitals but not from primary care settings. This study aimed to identify the frequency of MVC-related consultations in Australian general practices, explore the pharmacological management of health conditions related to those crashes, and investigate general practitioners’ (GPs) perceived barriers and enablers in managing these patients.

**Methods:**

Mixed-methods study. The quantitative component explored annual MVC-related consultation rates over seven years, the frequency of chronic pain, depression, anxiety or sleep issues after MVC, and management with opioids, antidepressants, anxiolytics or sedatives in a sample of 1,438,864 patients aged 16 + years attending 402 Australian general practices (MedicineInsight). Subsequently, we used content analysis of 81 GPs’ qualitative responses to an online survey that included some of our quantitative findings to explore their experiences and attitudes to managing patients after MVC.

**Results:**

MVC-related consultation rates remained stable between 2012 and 2018 at around 9.0 per 10,000 consultations. In 2017/2018 compared to their peers, those experiencing a MVC had a higher frequency of chronic pain (48% vs. 26%), depression/anxiety (20% vs. 13%) and sleep issues (7% vs. 4%). In general, medications were prescribed more after MVC. Opioid prescribing was much higher among patients after MVC than their peers, whether they consulted for chronic pain (23.8% 95%CI 21.6;26.0 vs. 15.2%, 95%CI 14.5;15.8 in 2017/2018, respectively) or not (15.8%, 95%CI 13.9;17.6 vs. 6.7%, 95% CI 6.4;7.0 in 2017/2018). Qualitative analyses identified a lack of guidelines, local referral pathways and decision frameworks as critical barriers for GPs to manage patients after MVC. GPs also expressed interest in having better access to management tools for specific MVC-related consequences (e.g., whiplash/seatbelt injuries, acute/chronic pain management, mental health issues).

**Conclusion:**

Chronic pain, mental health issues and the prescription of opioids were more frequent among patients experiencing MVC. This reinforces the relevance of appropriate management to limit the physical and psychological impact of MVC. GPs identified a lack of available resources (e.g. education, checklists and management support tools) for managing MVC-related consequences, and the need for local referral pathways and specific guidelines to escalate treatments.

**Supplementary Information:**

The online version contains supplementary material available at 10.1186/s12875-024-02421-5.

## Background

Globally, motor vehicle crashes (MVC) claim more than 1.35 million lives annually. The burden of crashes also extends to serious injuries, with around 50 million reported worldwide every year [1]. MVC can cause physical injuries, but also mental and social harm. Chronic pain, anxiety, depression, post-traumatic stress disorder or insomnia are examples of MVC-related effects that can influence social functioning, quality of life or working capacity [2, 3].

An Australian study using data from a major trauma centre and two hospitals found that 55% of patients involved in MVC reported moderate-severe anxiety symptoms before hospital discharge, and 6.5% continued to have those symptoms 6–8 months post-discharge [4]. In the US, an investigation using a national population-based survey found that adults reporting MVC had a higher prevalence of pain (55.8% vs. 44.0%) and anxiety (38.7 vs. 27.3%) than those not involved in MVC, even one year post-crash (51.3% vs. 42.9% and 35.2% vs. 26.1%, respectively) [5]. However, despite the high impact of MVC-related psychological conditions on people’s lives, they are usually neglected. There is a lack of published health outcome data after hospital discharge or among those treated in outpatient care, including recovery and rehabilitation [6, 7]. Previous studies highlighted the importance of understanding the impact of injuries and their psychological comorbidities in a timely manner to prevent difficulties in recovery, especially among groups more susceptible to MVC-related psychological conditions, such as older people [8] and women [9–11].

Many high-income countries have experienced reductions in MVC deaths and an increase in the likelihood of surviving [6]. Therefore, it is pertinent to explore how injuries and psychological consequences of MVC are managed in primary care to improve patient health and minimise the impact on healthcare costs, productivity and workplace disruption [12].

In Australia, the estimated costs related to MVC in 2015 was $22.2 billion, including $2.8 billion related to non-hospitalised injuries [13]. Most health data on MVC are based on insurance claims and hospital data [12, 14]. There is little information on the frequency of primary care consultations resulting from these crashes or the management of physical and psychological conditions among these patients in Australian general practice. In a national survey of Australian general practitioners (GPs), self-reported knowledge regarding the evidence-based diagnosis and management of MVC injuries was generally considered moderate to high. However, that study identified important gaps in GP’s knowledge when managing MVC-related conditions [15]. The authors highlighted that using electronic medical records in future studies would be a valuable data source to understand GPs’ behaviour, as self-reported adherence to guidelines may be distinct from actual clinical practice [15]. In addition, if more detailed information about current GP clinical practice for MVC management was available, resources such as targeted training, guidelines and local referral pathways could be developed. Also, early interventions, including follow-up consultations after discharge from hospital [8] or contact with health professionals who can provide psychological support [16, 17], could be designed to target the most vulnerable patient groups. All these actions might improve patient recovery and health-related quality of life.

This study aimed to identify (i) the frequency and temporal trend of consultations related to MVC in Australian general practice, (ii) the frequency of physical (chronic pain) and psychological conditions (depression, anxiety, sleep issues) related to these crashes recorded in patients’ medical records, and (iii) the management of these conditions including prescription of opioids, antidepressants, anxiolytics or sedatives. In addition, the study aimed to identify barriers perceived by GPs when managing patients after MVC and their preferences for educational resources to support their clinical practice.

## Methods

This study used a sequential explanatory mixed-methods approach [18]. In the first stage, data from electronic medical records of more than 1.4 million patients from 402 general practices across Australia included in the MedicineInsight database were analysed [19]. In the second stage, we examined GPs’ responses to an online survey about the findings of the quantitative analysis and their perspectives on managing patients after MVC. Details are described below.

### Stage 1 - quantitative component

#### Data extraction

We used a large national general practice database (MedicineInsight) that includes de-identified medical records of patients of all ages in all Australian states and regions. Routinely collected data includes sociodemographic and clinical data (e.g., diagnoses, reasons for consultation, immunisations, blood pressure, laboratory results, prescribed medications) [19]. We included data from all ‘regular’ patients aged 16 + years who had consultations between January 2012 and December 2018. We defined regular patients for each year of interest as those having at least one consultation in the year of interest, one in the previous year and one the year after (e.g., regular patients in 2017 had at least one visit in each of 2016, 2017 and 2018). Using this definition, we ensured that patients included in the sample would have available data on the exposure and outcomes. To improve data quality, only practices with consistent data provision (gaps in data provision lower than six weeks in the last two years and a ratio between the minimum and maximum number of annual consultations between 2011 and 2018 lower than five) were included.

### Outcomes

#### MVC consultation rates

As patients’ medical data can be recorded by using medical vocabulary coding systems or free text, we developed a data extraction algorithm including MVA (Motor Vehicle Accident) or MCA (Motor Car Accident) codes and free text, such as accident or crash and motor or vehicle or road or bike or traffic (Supplementary Table 1) and searched different fields of the MedicineInsight database (i.e. reason for encounter, diagnosis, reason for prescription), following recommendations for data extraction of routinely collected data [20]. We then calculated MVC-related consultation rates for each year from 2012 to 2018 among all consultations in each year. For this analysis, we used the total number of consultations as the denominator.

Analyses about the diagnosis and management of health conditions used the total number of patients as the denominator. These analyses only considered ‘incident’ MVC in 2017 (i.e. a patient consulted for MVC in 2017 but not in previous years).

#### Diagnosis of physical and mental health conditions related to MVC

The data extraction algorithm used standard medical terms, synonyms and misspellings of chronic pain, depression, anxiety or sleep issues, considering different fields of the database. Further details have been published elsewhere [21, 22]. We only included patients ‘at risk’ of developing these conditions between 2017 and 2018 (i.e. patients with any of these health diagnoses before 2017 were excluded). To analyse the relationship between MVC and these medical diagnoses, we calculated the proportion of patients with or without an ‘incident’ MVC in 2017 who had a ‘recent’ diagnosis of chronic pain, depression, anxiety or sleep issues (i.e., diagnosis in 2017 or 2018).

#### Pharmacological management of health conditions related to MVC

To explore the pharmacological management of these health conditions, we searched for prescriptions of opioids, antidepressants, anxiolytics and sedatives approved for use in Australia by the Therapeutic Goods Administration (www.tga.gov.au). Supplementary Table 2 lists medications included in the study. Further details are available in previous publications [21–23]. Again, we only included patients ‘at risk’ of being prescribed these medications in 2017 or 2018 (i.e. patients who received these medications in 2016 were excluded). We then calculated the proportion of patients with or without an incident MVC who received any of these prescriptions in 2017 or 2018 (i.e. ‘recent’ prescription).

### Covariates

Covariates included in the analyses were related to patient and practice characteristics. Patients’ characteristics included sex (male, female), age group (16–24, 25–34, 35–49, 50–64, 65–74, 75 + years), and the Index of Relative Socioeconomic Advantage and Disadvantage (IRSAD) quintiles based on residential postcode. IRSAD is a macroeconomic indicator of relative economic and social advantage/disadvantage position within an area compared to the rest of the country [24]. A higher IRSAD quintile indicates a more advantaged area. Practice characteristics included state (New South Wales, Victoria, Queensland, Western Australia, Tasmania, South Australia, Australian Capital Territory, Northern Territory), rurality (major cities, inner regional, outer regional/remote/very remote areas), and IRSAD quintiles based on the practice postcode.

### Statistical analysis

MVC-related consultation rates (per 10,000 consultations), overall and according to sociodemographic characteristics, were calculated for each year of the study (from 2012 to 2018). Consultation rates according to patient characteristics were adjusted for practice variables. Analyses also included a multiplicative interaction term between sex and age groups. We also estimate the average annual change in consultation rates using Poisson regression, considering general practices as clusters and using robust standard errors.

Logistic regression was used to analyse differences in the frequency of physical and mental health diagnoses and pharmacological treatment between patients with or without a record of MVC. All results were adjusted for patient (age, sex and IRSAD quintiles) and practice characteristics (remoteness and IRSAD quintiles). Results were obtained as adjusted odds ratios (OR_adj_) with their 95% confidence intervals, and were used to estimate adjusted predicted probabilities (i.e., adjusted proportions) through the command ‘margins’ in Stata for: 1) frequency of physical (chronic pain) and mental health conditions (depressive/anxiety symptoms and sleep issues), and 2) pharmacological management (opioids, antidepressants, anxiolytics and sedatives prescribing) of these conditions among patients with or without MVC. Results of the adjusted proportions and their 95% CI were then presented graphically and in supplementary tables. All analyses were performed in the statistical software Stata 16.1 (StataCorp, Texas, USA), using robust standard errors and considering the clustering of patients within the practices, and inverse probability weighting of being consulted.

### Stage 2 – qualitative component

The second part of this study included a qualitative content analysis (ca.) [25] of responses from GPs to an online survey about the quantitative results of the first part of the study and their experiences and attitudes to managing patients after MVC. The main objective was to identify current barriers and enablers for GPs managing patients after MVC and their preferences for professional education and support on this topic.

The online survey comprised 12 specific questions about managing MVC-related conditions (eight open-ended, free text and four closed-ended questions), three questions about their interest in receiving educational modules or resources related to managing patients with MVC, and six demographic questions (Supplementary Table 3). This approach was used to understand respondents’ perceived needs, interests or concerns, which may inform professional organisations about the challenges that GPs face when managing MVC-related conditions and inform the development of appropriate educational and support resources. For each open-ended question, all text responses were read as a whole to gain researcher immersion. Next, data was read word for word to generate initial codes, highlighting the exact words that captured key concepts. Researchers then developed a scheme that sorted codes into larger categories organised and labelled into meaningful clusters, with data then coded and counted using this coding scheme. Two experienced researchers (C.B. and E.H) conducted this content analysis to gain consensus about the dominant group of codes identified in the data [25].

## Results

### Quantitative analyses

**Fig. 1:**
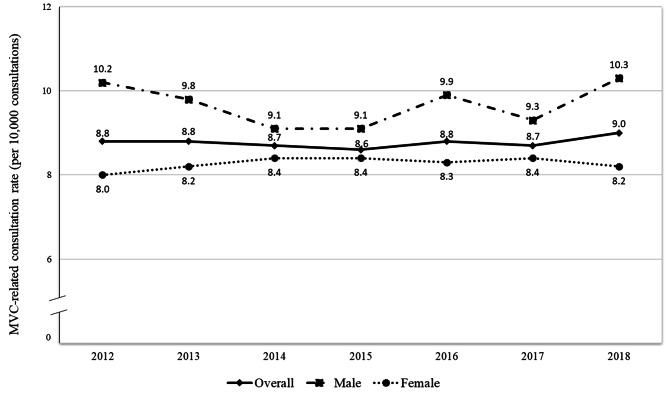
Temporal trend in MVC-related consultation rate (per 10,000 consultations, adjusted results) by sex in Australian general practice. Data on MVC recorded from 2012 to 2018

A total of 1,438,864 patients and 46,273,189 consultations from 2012 to 2018 were included in our study. Figure 1 shows that overall MVC-related consultation rates remained relatively stable over the years, ranging from 8.6 to 9.0 per 10,000 consultations yearly (average annual change: 1.1%, 95%CI: -0.7,2.9). Supplementary Table 4 shows that MVC-related consultation rates were higher among younger patients compared to older groups, and among patients from major cities compared to inner regional or remote areas. However, there were no evident differences according to patient or practice IRSAD quintiles. MVC consultation rates were higher among men than women in all investigated years. Additional analyses showed a different pattern in the sexes shaped by age: younger males had higher consultation rates than females, but the sex difference disappeared from the age of 50 years onwards (Supplementary Fig. 1).

**Fig. 2:**
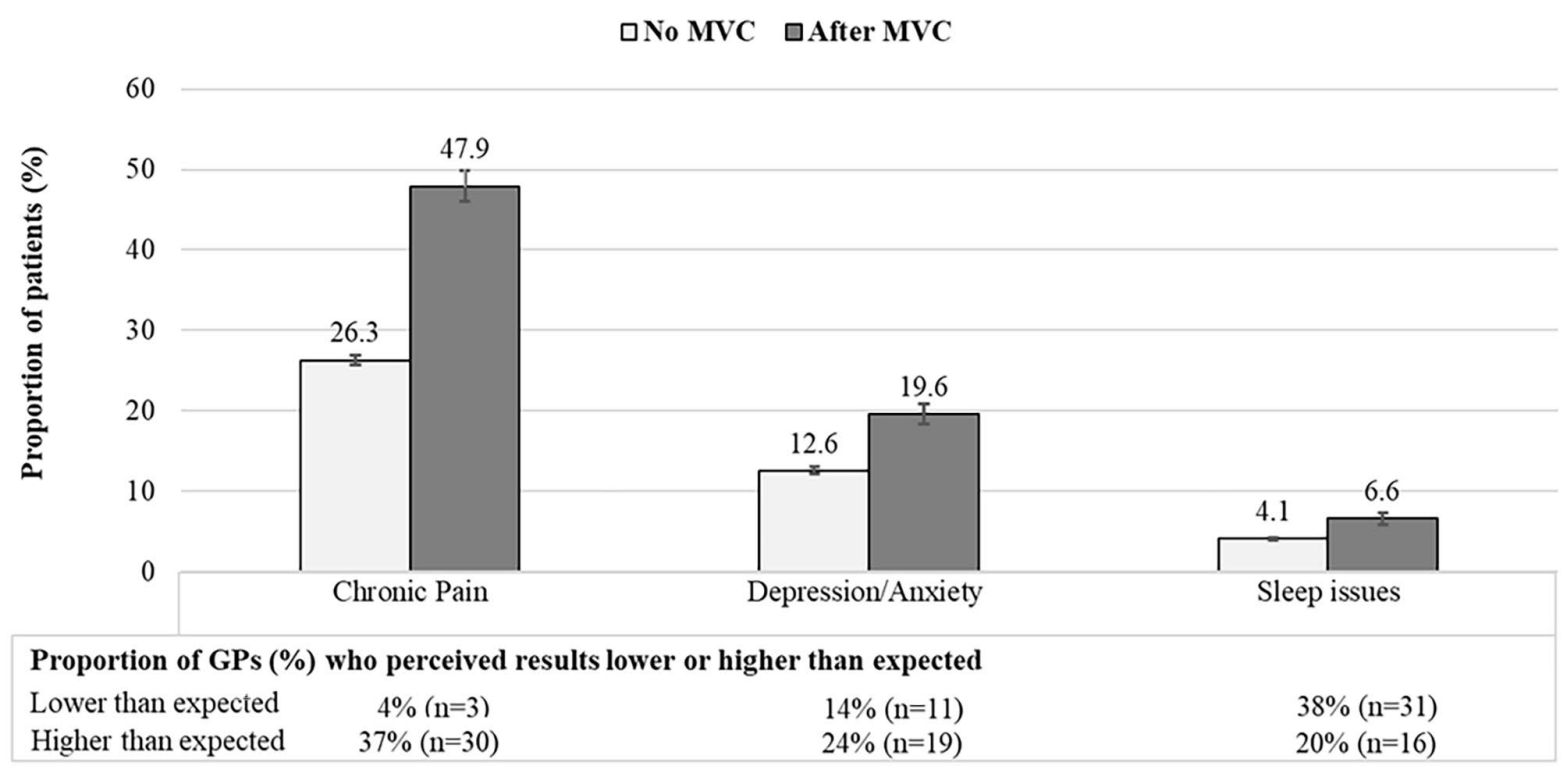
Proportion (%, adjusted results) and 95% CI (vertical lines) of regular patients in Australian general practice with a diagnosis in 2017/2018 (excluding those with the condition in 2016) among those with or without a record of MVC in 2017 (excluding those with previous MVC). The bottom table shows results of the qualitative component: proportion of the 81 respondent GPs who perceived these results lower or higher than expected (the remaining responded “as expected”)

Figure 2 shows that patients with an incident record of MVC in 2017 were more likely to get a ‘recent’ diagnosis of chronic pain (47.9% vs. 26.3%), depressive/anxiety symptoms (19.6% vs. 12.6%) or sleep issues (6.6% vs. 4.1%) than their peers. It is noted that almost half of patients with an MVC in 2017 had chronic pain and one out of every five people who suffered an MVC had depressive or anxiety symptoms.

Regarding medication prescribing, in general all investigated medications were more frequently prescribed to those with MVC than their peers, especially opioids (19.6%, 95%CI 18.1;21.2 vs. 8.9%, 95%CI 8.5;9, Supplementary Table 5). Additional analysis of prescribing by sociodemographic characteristics demonstrated that among patients with MVC, opioids and sedatives were more frequently prescribed for men than women, contrary to what was observed among those without MVC. The distribution of prescribing according to other characteristics was similar among patients with or without a record of MVC. For example, antidepressants and anxiolytics were more likely prescribed to women, opioids and sedatives to older age groups, antidepressants to younger age groups, and most medications were more frequently prescribed to those living in the most disadvantaged areas.

**Fig. 3:**
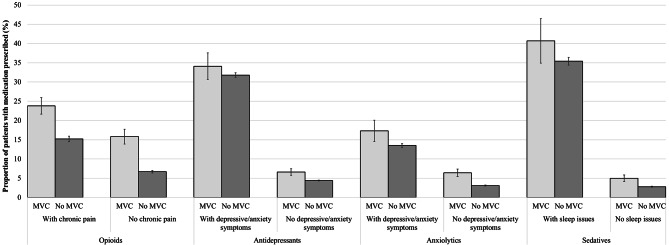
Proportion of regular patients in Australian general practice with medication prescribed among those with or without an incident MVC in 2017. Adjusted results stratified according to the presence or not of physical or psychological health conditions

Figure 3 analyses the prescribing patterns associated with the diagnosis of chronic pain, depressive/anxiety symptoms or sleep issues among patients with or without an MVC. The prescription of sedatives for patients with sleep issues, and antidepressants or anxiolytics for patients with depression/anxiety symptoms showed no clinical differences among patients with or without MVC. However, those with MVC and diagnosis of chronic pain had a much higher frequency of opioid prescribing than their peers (23.8% 95%CI 21.6;26.0 vs. 15.2%, 95%CI 14.5;15.8). In addition, those with a record of MVC without a diagnosis of chronic pain also had a high proportion of opioid prescribing than their peers with no MVC (15.8%, 95%CI 13.9;17.6 vs. 6.7%, 95% CI 6.4;7.0).

### Content analysis of survey responses

Of 81 GP respondents to the online questionnaire, 57% were female, 23% were located in a rural/remote general practice, 33% were less than 35 years, 54% were between 35 and 54 years, and 12% were 55 years or over. Around 72% of respondent GPs were not surprised about the frequency with which GPs see patients because of MVC, but a lower proportion responded that the diagnosis of chronic pain, depressive/anxiety symptoms and sleep issues after MVC were as expected (59%, 63% and 43%, respectively). Figure 2 shows that 37% and 24% of respondent GPs found the diagnosis of chronic pain and depression/anxiety among those with MVC higher than expected, respectively. On the other hand, more than one-third (38%) of the respondents found the diagnosis of sleep issues lower than expected.

GPs identified three main groups of factors as barriers to the management of MVC (Table 1). The first group related to the *Specific features of MVC presentations* (ca. 54), including (i) barriers related to delays in MVC presentations in a primary care setting, (ii) challenges for patients’ recollecting and lack of records tied to the MVC event, (iii) complexity of MVC trauma, (iv) protracted time commitments required to manage MVC related conditions and patient expectations, and (v) motivations and understanding of MVC treatment and recovery.

The second group of factors commonly identified as hindering care of patients after MVC related to *Requirements of Insurance*, (ca. 40), including (i) reporting burden, constraints related to approvals and payment, and (ii) the impact of insurance and legal cases on the recovery and expectations of patients. This group represents the multifaceted aspects and intricacies involved in insurance policies and procedures, including differences across Australian states. For instance, a compulsory third party motor vehicle insurance claim could be outside the scope of the Australian universal health insurance scheme (Medicare).

The third main group of factors identified related to *Limited access to allied health support* (ca. 19), including physiotherapy, imaging and psychological services. Less commonly identified barriers included the lack of relevant guidelines for treating MVC-related conditions and the lack of appropriate Medicare systems and remuneration commensurate with the time and complexity required by a GP to effectively treat patients after MVC.

**Table 1:** Main groups of factors identified by 81 GPs as barriers in managing patients after MVC

Main factors	Content Analysis (ca.)
**Specific features of MVC presentations** - Delays in MVC presentations in primary care - Challenges for patients’ recollecting and lack of records tied to MVC - Complexity of MVC trauma - Protracted time commitments required to manage MVC conditions - Motivations and understanding of MVC treatment and recovery	**ca. 54**
**Requirements of Insurance** - Reporting burden, constraints related to approvals and payment - Impact of insurance and legal cases on recovery and expectations	**ca. 40**
**Limited access to allied health support**	**ca. 19**
**Processes**	ca. 6
**Payment**	ca. 6
**Lack of guidelines**	ca. 4

Overall, there was widespread interest expressed by GPs (83% of respondents) to engage with educational modules and access resources to support their role in MVC treatment. As described in Table 2, a common area of interest was education and resources about referral and management pathways, including decision frameworks, flow charts and case studies for MVC-related treatments. Reference was made to a concussion protocol [26] as an exemplar that could be adapted for other MVC-related conditions. There was also widespread interest in educational resources about managing specific MVC-related conditions, including pain (acute and chronic) and whiplash, and structured approaches to MVC assessment and review.

**Table 2:** Educational areas of interest among 81 respondent GPs

Educational areas of interest	Content Analysis (ca.)
**Guidelines, pathways(referrals and management**) - Guidelines to escalate treatment/ specific to conditions/return to work/ indications for imaging/state specific - Referral pathways - Specific info about potential injuries based on speed and mechanism - Decision frameworks, flow charts and case studies - Protocol like one for concussion	**ca. 26**
**Education on managing common MVC related conditions** - Pain (acute/ chronic) - Whiplash, Cxspine strain, headaches - Seatbelt injuries - Ptsd, sleep, mental health - Fracture/ nonfracture - Concussion - Long term issues/ Their likely duration	**ca. 22**
**Assessment and management processes** - Structured approach to assessment - Checklists to ensure nothing missed in assessment - How to conduct a review, timelines	ca. 12
**Managing medication**	ca. 9
**Psychological support**	ca. 7
**Medico legal reports**	ca. 8
**Patient resources**	ca. 7
**Funding**	ca. 4

GPs most commonly preferred online modes of delivery for educational resources on this topic. Among multiple response choices, 62% of GPs indicated an interest in webinars, 51% websites and 41% short videos (up to 3 min) (Supplementary Fig. 2). GPs also suggested alternative options such as Continuing Professional Development (CPD) accredited modules, small group learning/peer-led learning, interactive Web-based learning, booklet, podcast, and information incorporated into GP software for patient handouts.

## Discussion

Our novel mixed-methods study analysing a nationwide general practice database and GPs attitudes regarding MVC-related consultations, health conditions and management in Australia have some key findings. MVC consultation rates remained stable between 2012 and 2018. The frequency of chronic pain, depression/anxiety and sleep issues, and the prescription of opioids was higher in patients with a record of MVC than those without it. However, a record of MVC did not seem to affect sedative prescribing for patients with sleep issues, or antidepressant and anxiolytic prescribing for those with depression/anxiety. Qualitative analyses identified the lack of clinical guidelines, local referral pathways and decision frameworks as key challenges for GPs when managing patients after MVC. GPs also expressed interest in having better access to management tools for specific MVC-related conditions, such as acute/chronic pain, whiplash, seatbelt injuries and mental health issues.

According to a report on the cost of road crashes in Australia based on various data sources, the number of MVC increased 3.9% from 2006 to 2015, with a decrease in fatalities of 24.3%, and a similar increase in hospitalised injuries (24.1%) [13]. However, there was no substantial increase in non-hospitalised injuries (only 0.8% change during the whole period), which may represent patients who visited general practice settings. It matches our results that MVC-related consultations remained stable over time (2012–2018). However, in terms of the magnitude of the problem, a previous Australian study with a nationally representative sample of around 3000 GPs (BEACH study, 2013–2016) found 0.047% of consultations, or an estimated 65,000 encounters per year related to the management of whiplash-associated disorders, the most common injury resulting from MVC [27], only half the number we found in this study (0.087% in 2017).

Additional analysis in our study found that MVC-related consultation rates were higher among younger patients compared to older groups, and patients from major cities compared to inner/outer/remote areas. Those findings were similar to results from the BEACH study [27], although they found higher MVC consultation rates among women than men, contrary to our findings. Nonetheless, another longitudinal study with novice drivers (aged 17–24 years) from New South Wales followed up for thirteen years found that young men had higher rates than young women for all crash types apart from crashes resulting in hospitalisations [28], which corroborates our results. The findings fit with the well-known increased risk of MVC among younger men compared to young women and older drivers [28–30].

Chronic pain was the most prevalent of the investigated conditions, with almost half of those involved in an MVC diagnosed with chronic pain after that event. This is in line with an international cohort study estimate that those who reported MVC had a 50% increase in the likelihood of having widespread chronic pain [31]. Given the prevalence of chronic pain in our study, it is unsurprising that GPs in this study specifically identified a need for education and support resources targeting the management of acute and chronic pain for patients after an MVC.

Differing from our results on the prevalence of depressive or anxiety symptoms (19.6%), the longitudinal study UQ SuPPORT with claimants from Queensland found that around 50% of participants (≥ 18 years) had a diagnosis of major depressive episode or generalized anxiety disorder or posttraumatic stress disorder in all periods observed (6, 12 and 24 months post crash) [32]. In contrast, a study with adult patients (18–64 years) of a major trauma centre and two hospitals in Melbourne showed that anxiety symptoms were present in 55% of patients in hospital after MVC, but this declined to 11.3% within 6–8 weeks post-crash and 6.5% within 6–8 months post-crash [4]. Disparities may be related to different methodologies, the severity of the MVC among patients attending hospital settings or primary care, the year of the study, and also response rates, with the former having 25.6% [32] and the latter 11.0% [4].

A key finding in this study was the high levels of opioid prescribing for those with chronic pain post-MVC. Further, even with no record of pain, those with MVC had higher rates of opioid prescribing. This fits with the findings of the general practice-based BEACH study, where authors found that opioids were the second most likely prescribed medication by GPs for patients with whiplash-associated disorders after simple analgesics. Opioids and opioids containing compound analgesics were prescribed at a rate of 21.8 per 100 whiplash-associated disorder problems. Non-recommended drugs for managing those injuries, including anxiolytics, anti-convulsants and antidepressants were used at a rate of 11.8 per 100 whiplash-associated disorder problems, representing 21.7% of all medications prescribed for the investigated patients [27].

Given that clinical practice guidelines stress the need for a cautious approach to prescribing opioids for whiplash-associated disorders [33] and for non-cancer chronic pain [34], the high rates of opioid prescribing reported here highlight the need to promote alternative treatment pathways. It is particularly relevant for older patients who were more frequently prescribed opioids and those living in disadvantaged areas who were more frequently prescribed medications after MVC. Moreover, GPs identified the lack of access to systems that facilitate allied health services such as physiotherapy and psychology, and the lack of local referral pathways as crucial barriers to the management of MVC-related conditions. These challenges may partly explain the current patterns of opioid prescribing tied to MVC. Another study carried out with GPs in Australia also found barriers related to the referral of patients after MVC for allied health services, such as out-of-pocket costs and long waiting lists [15].

In our study, GPs expressed interest in having better access to educational material for specific MVC-related conditions, such as acute/chronic pain, whiplash, seatbelt injuries and mental health issues. Despite the relatively infrequent occurrence of consultations for MVC compared to other conditions, GPs appear to be motivated to seek education due to either insufficient resources or challenges in accessing existing ones. Indeed, a previous Australian study on the effect of an online education program on GPs’ knowledge of whiplash guidelines improved by more than 20% among 57.2% of investigated GPs [35]. The authors mentioned the benefit of using online tools to reach GPs in rural and remote areas, and expected that results would motivate the development and implementation of clinical guidelines for other conditions [35]. However, according to our findings, GPs still require specific MVC guidelines. An investigation of the adoption and use of the whiplash guidelines showed that despite evidence of health professional practices being compatible with recommendations, there is also evidence of non-recommended practices that might lead to poor health outcomes [36]. Indeed, a paper with patients involved in minor transport-related injuries found most of them reported poor quality of care received from GPs [7], highlighting a need for more support, education and training for healthcare providers. It is essential a collaboration among professional and regulatory organisations to successfully deliver information on available resources and translate recommendations to clinical practice, particularly because of the challenges posed by differences across Australian states.

To provide feedback to respondent GPs, the research team searched for relevant resources currently available in Australian settings. This included sources such as professional organisations, Federal and state-based Return to Work/ WorkCover organisational websites, and Health Pathways provided by Primary Health Networks, funded by the Australian government. However, few open-access resources were found to support GPs in managing patients affected by a MVC, especially at a national level. The few resources that we found included: a guideline [33] and a webpage (mywhiplash.com.au) for the management of acute whiplash associate disorders for the first twelve weeks following an MVC, including a flowchart with assessment tools and recommended treatments; the website ‘Return to Work SA’ (https://www.rtwsa.com/), which provides open access tools and resources, such as online courses, potential pathways, webinars and handouts for the management of work-injured patients, which can also be used for patients injured in MVC; and Health Pathways for managing pain, mental health conditions, deprescribing, but nothing directly related to MVC, except for whiplash.

### Strengths and limitations

This study has strengths, such as the large nationwide general practice sample from all Australian states and geographic regions, providing an accurate estimate of GPs management of patients involved in MVC. To be able to directly link the diagnosis or prescribing with the occurrence of an MVC, we excluded from our analyses patients with diagnoses previous to the MVC, as well as those with previous prescriptions of any investigated medication. The study has also limitations, such as the fact that some MVC-related terms and diagnoses were based on free text, which may have resulted in underestimation of number of consultations and health conditions related to MVC. However, we included in the algorithm a variety of terms identified in the literature, as well as synonyms and misspellings for improving data extraction. Although we have not validated the MVC algorithm, previous algorithms used in the MedicineInsight database showed good to excellent accuracy on identifying health conditions, including depression and anxiety [37].

## Conclusion

Chronic pain, mental health issues and the prescription of opioids were more frequent among patients with MVC, reinforcing the importance of appropriate management of these patients to limit the physical and psychological impact of MVC. GPs identified a lack of available resources for managing MVC-related conditions and the need for local referral pathways and specific guidelines to escalate treatments. Collaborative efforts between GPs, professional organisations and researchers might focus on creating or improving dissemination of accessible and usable resources to support the clinical management of MVC-related chronic pain, mental health issues and prescription of opioids to improve health care for patients affected by MVC.

### Electronic supplementary material

Below is the link to the electronic supplementary material.

Supplementary Material 1

Supplementary Material 2

Supplementary Material 3

Supplementary Material 4

Supplementary Material 5

Supplementary Material 6

Supplementary Material 7

## Data Availability

The data that support the findings of this study are available from The Australian Commission on Safety and Quality in Health Care but restrictions apply to the availability of these data, which were used under license for the current study, and so are not publicly available. Data are however available from The Australian Commission on Safety and Quality in Health Care if approval is granted by the MedicineInsight Data Governance Committee. Data access enquiries can be directed to QUMprogram@safetyandquality.gov.au.

## Abbreviations

CI: Confidence Interval
GP: General Practitioners
IRSAD: Index of Relative Socioeconomic Advantage and Disadvantage
MVC: Motor Vehicle Accidents
MVC: Motor Vehicle Crashes
