# Supplementary material for: Management of physical and psychological trauma resulting from motor vehicle crashes in Australian general practice: a mixed-methods approach
Source: BMC Prim Care. 2024 May 16;25:167. doi: 10.1186/s12875-024-02421-5 (PMC11100075; doi:10.1186/s12875-024-02421-5)
Supplement: Supplementary file 1 — Supplementary Material 1 [file 12875_2024_2421_MOESM1_ESM.docx]

| **Supplementary table 1. Terms used to identify MVC-related consultations** | |
| --- | --- |
|  |  |
| **Term** | N* |
| MVC OR MCA | 120,178 |
| (motor OR road OR traffic OR vehicle OR car OR bike) AND (accident OR crash) | 55,251 |
| whiplash | 24,625 |
| seat belt injury | 83 |

*The same consultation can present more than one term. Misspellings were also included.
