# Supplementary material for: Management of physical and psychological trauma resulting from motor vehicle crashes in Australian general practice: a mixed-methods approach
Source: BMC Prim Care. 2024 May 16;25:167. doi: 10.1186/s12875-024-02421-5 (PMC11100075; doi:10.1186/s12875-024-02421-5)
Supplement: Supplementary file 2 — Supplementary Material 2 [file 12875_2024_2421_MOESM2_ESM.docx]

| **Supplementary table 2. List of medications included in the study.** |
| --- |

| **Antidepressants** | **Opioids** | **Anxiolytics** | **Sedatives** |
| --- | --- | --- | --- |
| **SSRI** (selective serotonin reuptake inhibitors): | buprenorphine | alprazolam | flunitrazepam |
| citalopram | codeine | bromazepam | midazolam |
| escitalopram | fentanyl | buspirone | nitrazepam |
| fluoxetine | hydromorphone | clobazam | temazepam |
| fluvoxamine | morphine | clonazepam | zolpidem |
| paroxetine | oxycodone | diazepam | zopiclone |
| sertraline | tapentadol | lorazepam |  |
| **SNRI** (selective noradrenaline reuptake inhibitors): | tramadol | oxazepam |  |
| desvenlafaxine |  |  |  |
| duloxetine |  |  |  |
| venlafaxine |  |  |  |
| **MAOI** (monoamine oxidase inhibitors): |  |  |  |
| moclobemide |  |  |  |
| phenylzine |  |  |  |
| tranylcypromine |  |  |  |
| **TAC** (tricyclic antidepressants): |  |  |  |
| amitriptyline |  |  |  |
| clomipramine |  |  |  |
| dothiepin |  |  |  |
| doxepin |  |  |  |
| imipramine |  |  |  |
| nortriptyline |  |  |  |
| trimipramine |  |  |  |
| **Others:** |  |  |  |
| agomelatine |  |  |  |
| bupropion |  |  |  |
| mianserin |  |  |  |
| mirtazapine |  |  |  |
| reboxetine |  |  |  |
| vortioxetine |  |  |  |
