# Supplementary material for: Management of physical and psychological trauma resulting from motor vehicle crashes in Australian general practice: a mixed-methods approach
Source: BMC Prim Care. 2024 May 16;25:167. doi: 10.1186/s12875-024-02421-5 (PMC11100075; doi:10.1186/s12875-024-02421-5)
Supplement: Supplementary file 3 — Supplementary Material 3 [file 12875_2024_2421_MOESM3_ESM.docx]

| **Supplementary table 3. Questions in the online survey answered by general practitioners.**   \| 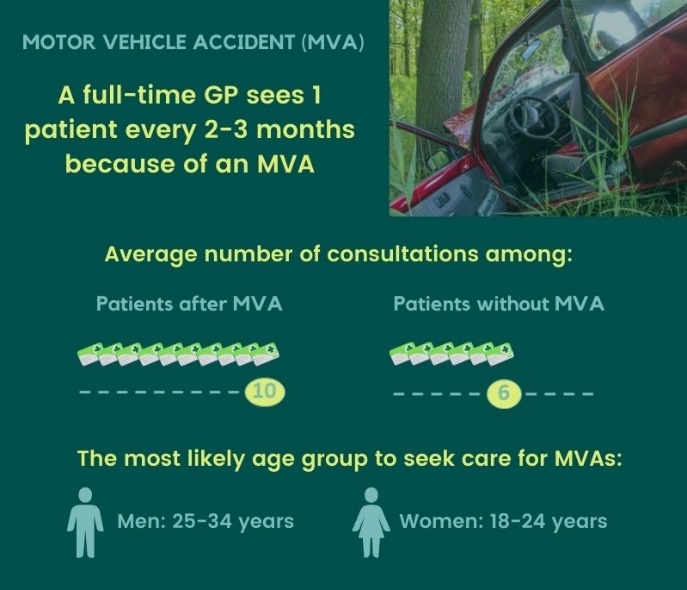  1. Do any of these findings surprise you?   - Yes - No \| \| --- \| \| 2. If yes, which are surprising and why? \| \| 3. What are the main factors that have hindered/would hinder you when managing patients after MVAs? \| \| 4. What are the main factors that have helped/would help you when managing patients after MVAs? \| \| 5. Looking at only those without sleep issues before an MVA, we found that 7% of patients had sleep issues after an MVA, compared to 4% of the overall patient population. Is this proportion what you would expect?   - It is lower than expected - It is higher than expected - It is as expected \| \| 6. What treatments would you consider for patients presenting with sleep issues after an MVA? \| \| 7. Looking only at those without depression and/or anxiety before the MVA, we found that 20% of patients had depression and/or anxiety after an MVA, compared to 13% of the overall patient population. Is this proportion what you would expect?   - It is lower than expected - It is higher than expected - It is as expected \| \| 8. What treatments would you consider for patients presenting with depression and/or anxiety after an MVA? \| \| 9. Looking only at those without chronic pain before the MVA, we found that 48% of patients had chronic pain after an MVA, compared to 27% of the overall patient population. Is this proportion what you would expect?   - It is lower than expected - It is higher than expected - It is as expected \| \| 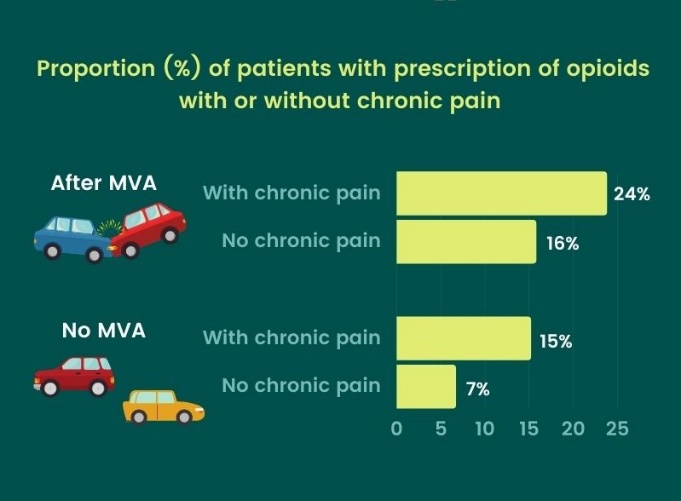  10. What treatments would you consider for patients presenting with chronic pain after an MVA? \| \| 11. In what situations would you prescribe opioids after an MVA? \| \| 12. As the figure shows, amongst patients who had an MVA, opioids were prescribed for 24% of those who did report chronic pain and 16% who did not report chronic pain.Why do you think GPs prescribe opioids for patients after an MVA, even if they do not report chronic pain? \| \| 13. Would you be interested in educational modules or resources about the management of patients after an MVA? If yes, what information would you like to receive? \| \| 14. If yes, how should these be delivered? Select all that apply.   - Email - Online flyer - Printed flyer - Short video (up to 3 minutes) - Long video (10-15 minutes) - Website - None of them - Other (please tell us about alternative options) \| \| 15. Are there any other comments you would like to make regarding GPs managing patients after an MVA that we have not covered already? \| \| 16. In a typical year, approximately how many patients do you see because of an MVA? \| \| 17. What is your gender?   - Female - Male - Other \| \| 18. What is your age?   - Up to 34 years - 35 to 54 years - 55 years or over \| \| 19. How long have you been working as a GP? \| \| 20. Generally, how many hours a week do you work as a GP? \| \| 21. Where is your current practice located?   - Rural/Remote - Metropolitan \| |
| --- | --- | --- | --- | --- | --- | --- | --- | --- | --- | --- | --- | --- | --- | --- | --- | --- | --- | --- | --- | --- | --- |
