# Supplementary material for: Management of physical and psychological trauma resulting from motor vehicle crashes in Australian general practice: a mixed-methods approach
Source: BMC Prim Care. 2024 May 16;25:167. doi: 10.1186/s12875-024-02421-5 (PMC11100075; doi:10.1186/s12875-024-02421-5)
Supplement: Supplementary file 4 — Supplementary Material 4 [file 12875_2024_2421_MOESM4_ESM.docx]

**Supplementary table 4. MVC-related consultation rates by sociodemographic variables among regular patients* from 2012 to 2018.**

|  | **Total patients** | | **MVC-related consultation rate (per 10,000 consultations)** | | | | | |  | **Annual Change** | | | | |
| --- | --- | --- | --- | --- | --- | --- | --- | --- | --- | --- | --- | --- | --- | --- |
|  | **N** | **(%)** | **2012** | **2013** | **2014** | **2015** | **2016** | **2017** | **2018** | | **%** | 95% CI | | |
| **Overall** | 1,438,864 | 100.0 | 8.8 | 8.8 | 8.7 | 8.6 | 8.8 | 8.7 | 9.0 | | 1.1 | -0.7 | ; | 2.9 |
| **Sex** |  |  |  |  |  |  |  |  |  | |  |  |  |  |
| Male | 601,858 | 41.9 | 10.2 | 9.8 | 9.1 | 9.1 | 9.9 | 9.3 | 10.3 | | 1.3 | -1.4 | ; | 4.0 |
| Female | 836,140 | 58.1 | 8.0 | 8.2 | 8.4 | 8.4 | 8.3 | 8.4 | 8.2 | | 0.9 | -0.9 | ; | 2.8 |
| **Age** |  |  |  |  |  |  |  |  |  | |  |  |  |  |
| 16-24 | 194,375 | 10.8 | 18.7 | 17.7 | 17.3 | 15.5 | 18.4 | 17.8 | 17.0 | | -0.9 | -3.4 | ; | 1.7 |
| 25-34 | 236,585 | 13.1 | 16.0 | 17.1 | 15.9 | 14.7 | 16.4 | 15.7 | 15.9 | | -0.9 | -4.0 | ; | 2.3 |
| 35-49 | 412,805 | 22.9 | 13.8 | 14.1 | 14.7 | 14.0 | 14.3 | 15.1 | 14.8 | | 1.0 | -1.4 | ; | 3.6 |
| 50-64 | 462,241 | 25.6 | 8.9 | 9.4 | 9.6 | 10.7 | 10.6 | 10.7 | 11.7 | | 4.0 | 1.3 | ; | 6.9 |
| 65-74 | 301,870 | 16.7 | 4.6 | 4.4 | 4.1 | 4.8 | 5.0 | 4.7 | 4.2 | | -0.2 | -3.8 | ; | 3.5 |
| 75+ | 197,855 | 11.0 | 3.2 | 2.8 | 2.9 | 2.5 | 2.6 | 2.4 | 2.7 | | -2.7 | -8.1 | ; | 3.1 |
| **Patient IRSAD quintiles** |  |  |  |  |  |  |  |  |  | |  |  |  |  |
| Highest | 362,783 | 30.6 | 9.2 | 8.2 | 8.5 | 8.0 | 8.9 | 8.7 | 9.7 | | 1.1 | -1.6 | ; | 3.8 |
| Second highest | 242,707 | 17.0 | 7.8 | 7.9 | 9.4 | 8.6 | 8.6 | 8.6 | 9.2 | | 2.2 | -0.6 | ; | 5.0 |
| Intermediate | 328,329 | 27.7 | 7.9 | 8.8 | 8.9 | 8.6 | 8.0 | 8.1 | 8.7 | | 2.6 | -0.8 | ; | 6.0 |
| Second lowest | 233,416 | 19.7 | 10.1 | 9.5 | 8.6 | 9.8 | 9.9 | 9.8 | 9.7 | | 0.9 | -2.3 | ; | 4.3 |
| Lowest | 260,199 | 22.0 | 9.2 | 10.3 | 7.8 | 9.1 | 9.5 | 9.1 | 7.5 | | -1.3 | -7.3 | ; | 5.1 |
| **GP state** |  |  |  |  |  |  |  |  |  | |  |  |  |  |
| New South Wales | 508,424 | 35.3 | 7.4 | 7.2 | 6.7 | 7.2 | 7.6 | 7.0 | 7.3 | | 0.8 | -1.9 | ; | 3.5 |
| Victoria | 318,917 | 22.2 | 7.2 | 7.7 | 7.7 | 7.0 | 7.5 | 7.6 | 9.0 | | 2.7 | -1.7 | ; | 7.4 |
| Queensland | 199,765 | 13.9 | 6.6 | 5.6 | 7.0 | 6.1 | 6.7 | 6.9 | 7.1 | | 2.0 | -0.8 | ; | 4.9 |
| Western Australia | 173,393 | 12.1 | 14.3 | 14.0 | 12.3 | 11.9 | 12.5 | 13.9 | 12.4 | | -1.1 | -7.9 | ; | 6.3 |
| Tasmania | 146,222 | 10.2 | 10.9 | 10.9 | 11.1 | 12.1 | 9.7 | 9.7 | 9.5 | | -5.2 | -10.0 | ; | -0.2 |
| South Australia | 41,022 | 2.9 | 13.4 | 13.6 | 15.9 | 13.7 | 12.9 | 11.2 | 10.5 | | -4.6 | -8.1 | ; | -1.7 |
| Australian Capital Territory | 37,807 | 2.6 | 19.2 | 24.6 | 22.1 | 28.3 | 27.6 | 25.0 | 23.8 | | 1.4 | -4.9 | ; | 8.0 |
| Northern Territory | 13,314 | 0.9 | 4.8 | 2.1 | 3.7 | 2.8 | 4.0 | 4.7 | 6.1 | | 1.8 | -15.7 | ; | 23.0 |
| **GP Rurality** |  |  |  |  |  |  |  |  |  | |  |  |  |  |
| Major Cities | 864,680 | 60.1 | 9.9 | 10.2 | 10.5 | 11.0 | 11.6 | 11.0 | 10.8 | | 0.9 | -1.0 | ; | 2.9 |
| Inner Regional | 378,897 | 26.3 | 7.4 | 7.4 | 7.2 | 6.5 | 6.4 | 6.5 | 7.5 | | 0.2 | -3.2 | ; | 3.8 |
| Outer regional/Remote | 195,287 | 13.6 | 6.7 | 5.5 | 3.9 | 3.8 | 3.3 | 4.0 | 4.3 | | -7.2 | -16.9 | ; | 3.7 |
| **GP IRSAD quintiles** |  |  |  |  |  |  |  |  |  | |  |  |  |  |
| Highest | 393,552 | 27.5 | 9.4 | 8.5 | 8.4 | 8.0 | 7.7 | 8.0 | 8.4 | | -0.3 | -2.9 | ; | 2.4 |
| Second highest | 230,785 | 16.1 | 10.4 | 11.0 | 10.5 | 10.8 | 9.9 | 9.6 | 10.0 | | -0.5 | -3.7 | ; | 2.8 |
| Intermediate | 330,587 | 23.1 | 7.1 | 7.3 | 7.4 | 7.6 | 7.8 | 8.2 | 8.4 | | 2.5 | -1.1 | ; | 6.2 |
| Second lowest | 207,904 | 14.5 | 8.6 | 9.8 | 9.5 | 9.1 | 10.2 | 9.8 | 9.4 | | -0.8 | -4.4 | ; | 2.8 |
| Lowest | 269,972 | 18.8 | 9.0 | 8.8 | 8.6 | 9.2 | 10.9 | 9.6 | 9.8 | | 0.0 | -7.4 | ; | 8.1 |
| ***Total consultations*** | *46,273,189* | | *4,551,529* | *5,204,489* | *5,908,750* | *6,647,884* | *7,327,173* | *8,040,366* | *8,592,998* | |  |  |  |  |

* Patients aged 16+ years who have at least one consultation in that year, the previous and the next year.
