# Supplementary material for: Management of physical and psychological trauma resulting from motor vehicle crashes in Australian general practice: a mixed-methods approach
Source: BMC Prim Care. 2024 May 16;25:167. doi: 10.1186/s12875-024-02421-5 (PMC11100075; doi:10.1186/s12875-024-02421-5)
Supplement: Supplementary file 5 — Supplementary Material 5 [file 12875_2024_2421_MOESM5_ESM.docx]

**Supplementary table 5. Proportion of regular patients* with opioids, sedatives, antidepressants, anxiolytics or sedatives prescribed in 2017/2018 (excluding patients prescribed these medications in 2016) among those with or without a record of MVC in 2017 (excluding those with any previous MVC)**

|  | **Opioids**  **(%)** | | **Antidepressants (%)** | | **Anxiolytics**  **(%)** | | **Sedatives**  **(%)** | |
| --- | --- | --- | --- | --- | --- | --- | --- | --- |
| **Variable** | MVC | No MVC | MVC | No MVC | MVC | No MVC | MVC | No MVC |
| **Sex** |  |  |  |  |  |  |  |  |
| Male | 20.4 | 8.8 | 10.3 | 6.9 | 6.9 | 3.8 | 7.9 | 3.6 |
| Female | 16.3 | 9.0 | 13.8 | 8.6 | 9.8 | 4.9 | 5.3 | 4.5 |
| **Age** |  |  |  |  |  |  |  |  |
| 16-24 | 12.6 | 4.8 | 13.9 | 9.7 | 7.4 | 2.8 | 3.3 | 1.9 |
| 25-34 | 17.4 | 6.8 | 14.4 | 9.0 | 9.7 | 4.6 | 6.2 | 3.0 |
| 35-49 | 16.9 | 8.0 | 13.1 | 8.7 | 9.3 | 5.2 | 7.4 | 3.8 |
| 50-64 | 18.8 | 8.9 | 10.5 | 7.1 | 9.1 | 4.5 | 6.4 | 4.5 |
| 65-74 | 23.2 | 10.3 | 10.5 | 6.2 | 7.5 | 4.2 | 11.4 | 4.8 |
| 75+ | 23.1 | 13.9 | 11.2 | 7.8 | 6.4 | 4.3 | 8.6 | 5.8 |
| **Patient IRSAD quintiles** |  |  |  |  |  |  |  |  |
| Most advantaged | 18.5 | 7.6 | 11.0 | 7.0 | 7.4 | 4.1 | 6.7 | 4.2 |
| Second most advantaged | 17.4 | 8.9 | 13.8 | 7.8 | 8.2 | 4.5 | 6.8 | 4.0 |
| Intermediate | 17.7 | 9.3 | 14.0 | 8.0 | 9.9 | 4.6 | 6.9 | 4.1 |
| Second most disadvantaged | 17.6 | 9.0 | 10.2 | 8.3 | 8.7 | 4.5 | 5.7 | 3.9 |
| Most disadvantaged | 18.8 | 9.9 | 13.7 | 8.7 | 10.0 | 4.6 | 9.1 | 4.2 |
| **GP state** |  |  |  |  |  |  |  |  |
| New South Wales | 17.0 | 8.5 | 11.6 | 7.8 | 8.0 | 4.0 | 6.6 | 3.8 |
| Victoria | 18.4 | 9.6 | 10.5 | 7.1 | 7.9 | 4.7 | 8.0 | 4.6 |
| Queensland | 17.3 | 8.9 | 13.2 | 8.3 | 11.7 | 4.9 | 4.5 | 4.2 |
| Western Australia | 20.0 | 9.0 | 17.1 | 8.7 | 11.1 | 4.7 | 8.9 | 4.4 |
| Tasmania | 19.3 | 8.8 | 12.3 | 8.5 | 6.7 | 4.7 | 6.9 | 3.8 |
| South Australia | 15.2 | 8.9 | 11.7 | 7.0 | 7.4 | 4.0 | 2.8 | 4.2 |
| Australian Capital Territory | 17.3 | 8.3 | 11.9 | 9.0 | 5.8 | 3.8 | 7.8 | 3.7 |
| Northern Territory | 25.3 | 10.1 | 13.6 | 6.7 | - | 2.9 | 9.3 | 2.9 |
| **GP Remoteness** |  |  |  |  |  |  |  |  |
| Major Cities | 17.0 | 8.5 | 12.1 | 7.6 | 8.1 | 4.4 | 6.2 | 3.9 |
| Inner Regional | 21.3 | 8.9 | 13.0 | 8.3 | 10.5 | 4.6 | 9.1 | 4.4 |
| Outer regional/Remote | 16.9 | 10.9 | 13.1 | 8.1 | 9.2 | 4.3 | 7.8 | 4.3 |
| **GP IRSAD quintiles** |  |  |  |  |  |  |  |  |
| Most advantaged | 17.0 | 7.6 | 12.5 | 7.3 | 9.7 | 4.7 | 7.6 | 4.6 |
| Second most advantaged | 15.7 | 8.9 | 11.8 | 8.2 | 9.5 | 4.7 | 9.4 | 4.5 |
| Intermediate | 20.0 | 9.1 | 11.7 | 8.0 | 7.5 | 4.3 | 5.3 | 3.9 |
| Second most disadvantaged | 17.5 | 9.2 | 11.4 | 8.2 | 7.6 | 4.2 | 6.6 | 3.7 |
| Most disadvantaged | 19.8 | 10.3 | 14.9 | 8.2 | 7.9 | 4.1 | 5.6 | 3.7 |
| **Total** | **19.6** | **8.9** | **12.0** | **7.9** | **8.5** | **4.4** | **7.3** | **4.1** |

* Patients aged 16+ years who have at least one annual consultation in 2016, 2017 and 2018.
