# Supplementary material for: Management of physical and psychological trauma resulting from motor vehicle crashes in Australian general practice: a mixed-methods approach
Source: BMC Prim Care. 2024 May 16;25:167. doi: 10.1186/s12875-024-02421-5 (PMC11100075; doi:10.1186/s12875-024-02421-5)
Supplement: Supplementary file 6 — Supplementary Material 6 [file 12875_2024_2421_MOESM6_ESM.docx]

**Supplementary figure 1. MVC-related consultation rate (per 10,000 consultations, adjusted results) and 95% CI (vertical lines) by sex and age group. Data on MVCs recorded from 2012 to 2018 in Australia general practice.**
