# Supplementary material for: Management of physical and psychological trauma resulting from motor vehicle crashes in Australian general practice: a mixed-methods approach
Source: BMC Prim Care. 2024 May 16;25:167. doi: 10.1186/s12875-024-02421-5 (PMC11100075; doi:10.1186/s12875-024-02421-5)
Supplement: Supplementary file 7 — Supplementary Material 7 [file 12875_2024_2421_MOESM7_ESM.docx]

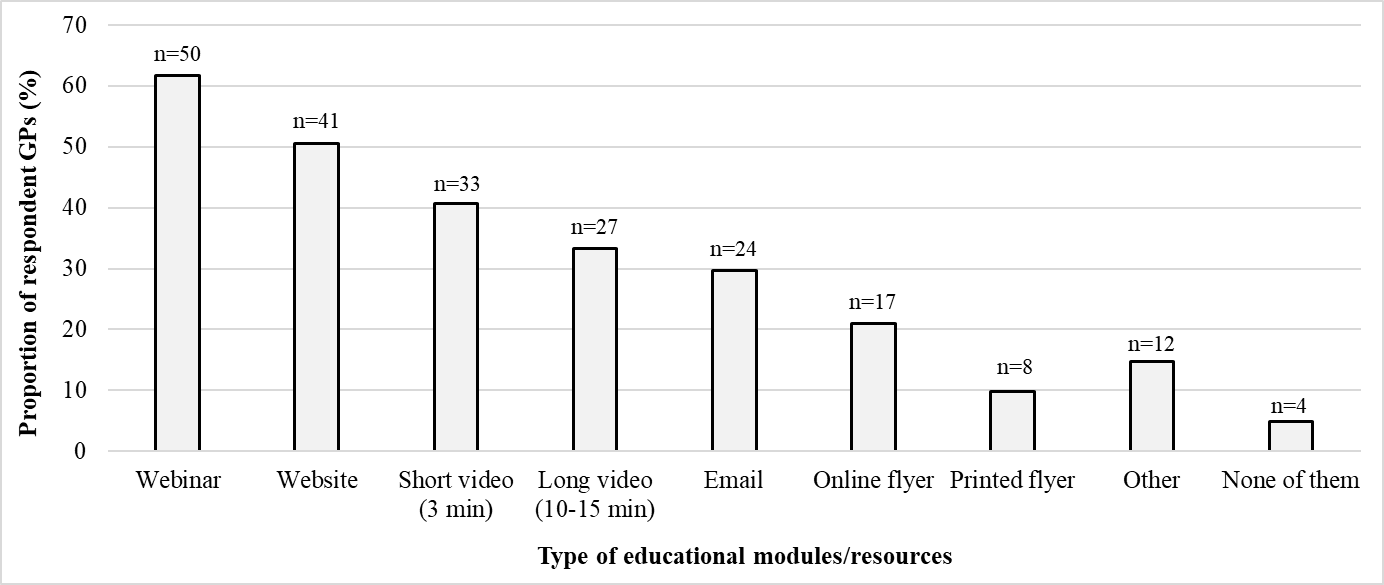


**Supplementary figure 2. Proportion (%) of GPs who were interested in receiving educational modules/resources on the management of patients after an MVC among all 81 respondents to the online survey. GPs could select multiple options.**
